# Polydopamine-Assisted Silver Nanoparticle Self-Assembly on Sericin/Agar Film for Potential Wound Dressing Application

**DOI:** 10.3390/ijms19102875

**Published:** 2018-09-21

**Authors:** Liying Liu, Rui Cai, Yejing Wang, Gang Tao, Lisha Ai, Peng Wang, Meirong Yang, Hua Zuo, Ping Zhao, Huawei He

**Affiliations:** 1 State Key Laboratory of Silkworm Genome Biology, Southwest University, Chongqing 400715, China; l3341345@email.swu.edu.cn (L.L.); taogang@email.swu.edu.cn (G.T.); als123@email.swu.edu.cn (L.A.); yangmeirong@email.swu.edu.cn (M.Y.); zhaop@swu.edu.cn (P.Z.); 2 College of Biotechnology, Southwest University, Chongqing 400715, China; cairui0330@email.swu.edu.cn (R.C.); modelsums@email.swu.edu.cn (P.W.); 3 College of Pharmaceutical Sciences, Southwest University, Chongqing 400715, China; zuohua@swu.edu.cn; 4 Chongqing Key Laboratory of Sericultural Science, Chongqing Engineering and Technology Research Center for Novel Silk Materials, Southwest University, Chongqing 400715, China

**Keywords:** polydopamine, silver nanoparticle, sericin, antimicrobial activity, cytocompatibility

## Abstract

Silver nanoparticles (AgNPs) are extensively applied for their broad-spectrum and excellent antibacterial ability in recent years. Polydopamine (PDA) has great advantages for synthesizing large amounts of AgNPs, as it has multiple sites for silver ion binding and phenolic hydroxyl structure to reduce silver ions to AgNPs. Here, we mixed sericin and agar solution and dried at 65 °C to prepare a sericin (SS)/Agar composite film, and then coated polydopamine (PDA) on the surface of SS/Agar film by soaking SS/Agar film into polydopamine solution, subsequently synthesizing high-density AgNPs with the assistance of PDA to yield antibacterial AgNPs-PDA- SS/Agar film. Scanning electron microscope (SEM), Fourier transform infrared spectroscopy (FT-IR) and X-ray diffraction (XRD) spectra indicated the successful synthesis of high-density AgNPs on the surface of PDA-SS/Agar film. PDA coating and AgNPs modification did not affect the structure of sericin and agar. Furthermore, water contact angle, water absorption and mechanical property analysis showed that AgNPs-PDA-SS/Agar film had excellent hydrophilicity and proper mechanical properties. Inhibition zone and growth curve assays suggested the prepared film had excellent and long-lasting antibacterial ability. In addition, it had excellent cytocompatibility on the fibroblast NIH/3T3 cells. The film shows great potential as a novel kind of wound dressing.

## 1. Introduction

The antibacterial ability of a material surface is crucial to inhibiting the growth of bacteria on and around the material, which has great potential in food packaging and biomedical application [1]. Recently, nanomaterials have received increasing interest for their specific properties and applications in different fields. Silver nanoparticle (AgNP) is a brilliant nanomaterial, as it has a broad inhibitive effect against a variety of bacteria and fungus [2]. Surface immobilization of AgNPs is one of the most effective ways to increase the antibacterial property of materials [3].

Silk sericin (SS) is a natural hydrophilic macromolecular protein derived from silkworm cocoon. Sericin makes up 25–30% of silkworm cocoon, and it wraps silk fibroin fiber with a continuous, viscous layer that helps the formation of cocoon [4]. Sericin is a polymer protein with a molecular weight ranging from 10 to over 300 kDa. Sericin has high contents of serine and aspartate, accounting for about 33.4% and 16.7%, respectively [4]. Serine and aspartic acid have strong polar side chains. Thus, sericin can easily copolymerize and blend with other macromolecules to produce biocompatible materials with enhanced properties [5,6]. Sericin is considered to be one of the skin’s important natural moisturizing factors due to its excellent hydrophilicity and hygroscopicity [7]. In addition, sericin has a great deal of excellent properties, such as biocompatibility, oxidation resistance, and anticoagulation [8]. The moisturizing property, the ability of promoting epithelial cell growth and oxidation resistance mean that sericin possesses great potential in biomedical applications. Silk-based materials have been attracting increasing interest for biomedical materials and tissue engineering applications in recent years. Chlapanidas et al. studied the biological properties of silk fibroin from 20 strains, and then picked the most promising strains in which sericin was developed for cosmetic and dermatological applications [9]. They also showed that sericin microspheres loaded with tumor necrosis factor-α (TNF-α) blockers contribute to the down-regulation of cytokines [10]. In addition, a great deal of research on sericin and silk fibroin-based biomaterials, including the use of silk fibroin microspheres as a promoting wound healing material or a local coagulant [11,12,13], and sericin as a natural carrier for drug delivery, has been documented [14,15,16]. However, sericin contains a large amount of random coil structures, resulting in the formation of amorphous and brittle sericin materials which are not suitable for biomaterial application [17]. Therefore, sericin is usually cross-linked or blended with other polymers to enhance its mechanical performance. Agar is a macromolecular polysaccharide with hydrophilic, biocompatible and biodegradable ability [18]. In our previous study, we developed sericin/agar composite film modified with AgNPs to expand the application of sericin-based biomaterials [19,20]. AgNPs are synthesized with the assistance of ultraviolet (UV) light irradiation. However, long-term exposure under UV light may cause damage to sericin. Also, the aggregation of AgNPs on sericin is a major drawback of the UV-assisted method, as well as other methods currently available. In addition, the high-density synthesis of AgNPs is another key issue to be considered in the preparation of AgNPs functionalized materials. Recently, kinds of polymers such as poly (vinyl pyrrolidone) and polyamide network have been used as three-dimensional substrates for high-density growth of AgNPs [21,22]. However, most polymers are hydrophobic and not suitable for biomedical application. Therefore, it is important to find a substance which could not only improve the density of AgNPs, but also increase the hydrophilicity of the material surface for biomaterial application. Dopamine (DA) is a small molecule, and is the main component of viscous proteins secreted by mussels and can self-polymerize to polydopamine (PDA) under alkaline conditions and adhere to almost any substrate [23,24]. PDA contains several hydrophilic groups, such as -OH, -COOH and -NH_2_ [25]. PDA coating is an effective method used in recent years to improve the hydrophilicity and biocompatibility of materials [26,27]. PDA can not only provide sites for metal ions binding, but also reduce silver ions to AgNPs with its phenolic hydroxyl groups. PDA has been proved to be non-toxic to cells [25,28]. Thus, PDA is a very promising candidate for AgNPs synthesis. The issue of whether PDA can produce biologically active dopamine in vivo has not been well addressed in the literature on PDA-modified materials. 

In this work, we utilized PDA to assist the synthesis of high-density AgNPs on the surface of PDA-SS/Agar film to yield AgNPs-PDA-SS/Agar film. Scanning electron microscopy (SEM), Fourier transform infrared spectroscopy (FT-IR), X-ray diffraction (XRD) confirmed the high-density synthesis of AgNPs on the surface of the blend film. In addition, the novel film exhibited excellent hydrophilicity and proper mechanical property. Antibacterial tests indicated that the fabricated film had excellent antibacterial activity against *Escherichia coli* (*E. coli*) and *Staphylococcus aureus* (*S. aureus*). Cell viability assay indicated the composite film had excellent cytocompatibility on the fibroblast NIH/3T3 cells. AgNPs-PDA-SS/Agar film shows great prospects in novel wound dressing, artificial skin, tissue engineering and antibacterial packaging. In addition, SS/Agar composite can be prepared into three-dimensional scaffold and gel materials to expand its application in bone repair and injectable hydrogel materials.

## 2. Results and Discussion

**Figure 1:**
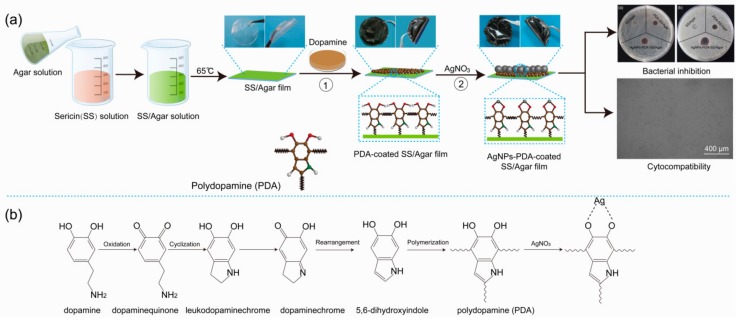
A diagram of the preparation of antibacterial AgNPs-PDA-SS/Agar film (**a**); schematic diagram of dopamine polymerization and AgNPs synthesis with the assistance of PDA (**b**).

Here, we used PDA to direct the synthesis of the antibacterial AgNPs on the surface of PDA-coated SS/Agar film. The principle and procedure are briefly shown in Figure 1. The procedure of the preparation of the films is shown in Figure 1a. Agar solution (2%, *w/v*) was added into sericin solution (2%, *w/v*) to become sericin/Agar mixture, and then dried at 65 °C to form SS/Agar composite film. Next, dopamine hydrochloride powder was dissolved in Tris-HCl buffer (pH 8.5) to become 2% (*w/v*) polydopamine solution. Then, SS/Agar film was immersed into dopamine solution to produce PDA-coated SS/Agar film. Furthermore, PDA-SS/Agar film was soaked in AgNO_3_ solution at room temperature for 4 h. The PDA layer acts as a secondary reaction platform, which can not only provide sites for metal ions binding, but also reduce silver ions to AgNPs with its phenolic hydroxyl groups to promote the synthesis of high-density AgNPs on the PDA-SS/Agar film. The prepared AgNPs-PDA-SS/Agar film was expected to have excellent antibacterial ability and cytocompatibility for wound dressing applications.

Figure 1b shows the mechanism of dopamine polymerization and AgNPs synthesis with the assistance of PDA. First, dopamine was oxidized to form dopamine quinone, and then dopamine quinone was converted to leukodopaminechrome by cyclization. Leukodopaminechrome was oxidized to form dopaminechrome. Finally, dopaminechrome was transformed to PDA by means of rearrangement and polymerization. Almost all dopamine can be converted to PDA under alkaline conditions. PDA contains active phenolic hydroxyl groups, which can react with silver ions to reduce them to AgNPs. 

### 2.1. Scanning Electron Microscope (SEM), Energy Dispersive Spectroscopy (EDS)

**Figure 2:**
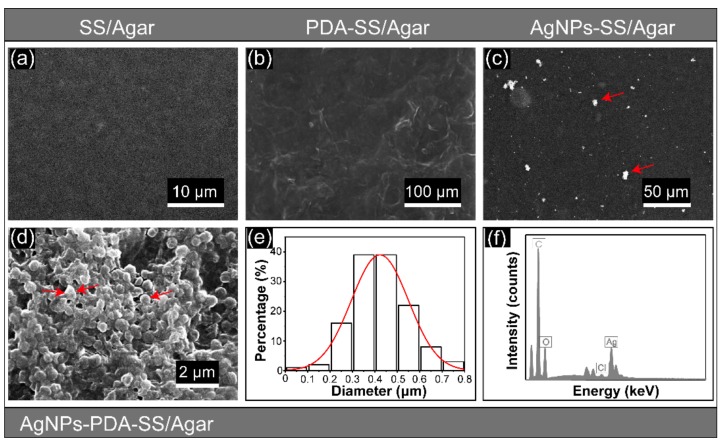
Surface morphologies of SS/Agar (**a**); PDA-SS/Agar (**b**); AgNPs-SS/Agar (**c**); AgNPs-PDA-SS/Agar films (**d**); (**e**) is the particle size analysis of (**d**); EDS spectrum of AgNPs-PDA-SS/Agar film (**f**). Red arrows indicate high-density AgNPs.

The SEM images of SS/Agar, PDA-SS/Agar, AgNPs-SS/Agar and AgNPs-PDA-SS/Agar films are shown in Figure 2. SS/Agar film exhibited a uniform and smooth surface (Figure 2a), indicating that sericin and agar were well blended. Figure 2b shows the surface of PDA-SS/Agar film, which was covered with a layer of ridge-like substance. SS/Agar film was soaked into PDA solution at room temperature for 12 h to ensure PDA coating on its surface. Thus, we deduced that the ridge-like substance covering the surface of SS/Agar film was the coated PDA. Figure 2c shows the SEM image of AgNPs-SS/Agar film without PDA. High-density AgNPs (marked by red arrows) were observed in the SEM image of AgNPs-PDA-SS/Agar film (Figure 2d). The number of AgNPs in Figure 2d were much greater than in Figure 2c, which indirectly indicates the existence of PDA on the SS/Agar film, and suggests that PDA could effectively promote the synthesis of large amounts of AgNPs. Particle size analysis revealed that the size of the synthesized AgNPs was concentrated at 300–500 nm (Figure 2e). Most of the particles were spherical in shape. In some cases, AgNPs seemed to be merged, as the density of the particles was too high (Figure 2d). Furthermore, EDS confirmed the presence of silver in the AgNPs-PDA-SS/Agar composite film (Figure 2f).

### 2.2. Fourier Transform Infrared Spectroscopy (FT-IR)

**Figure 3:**
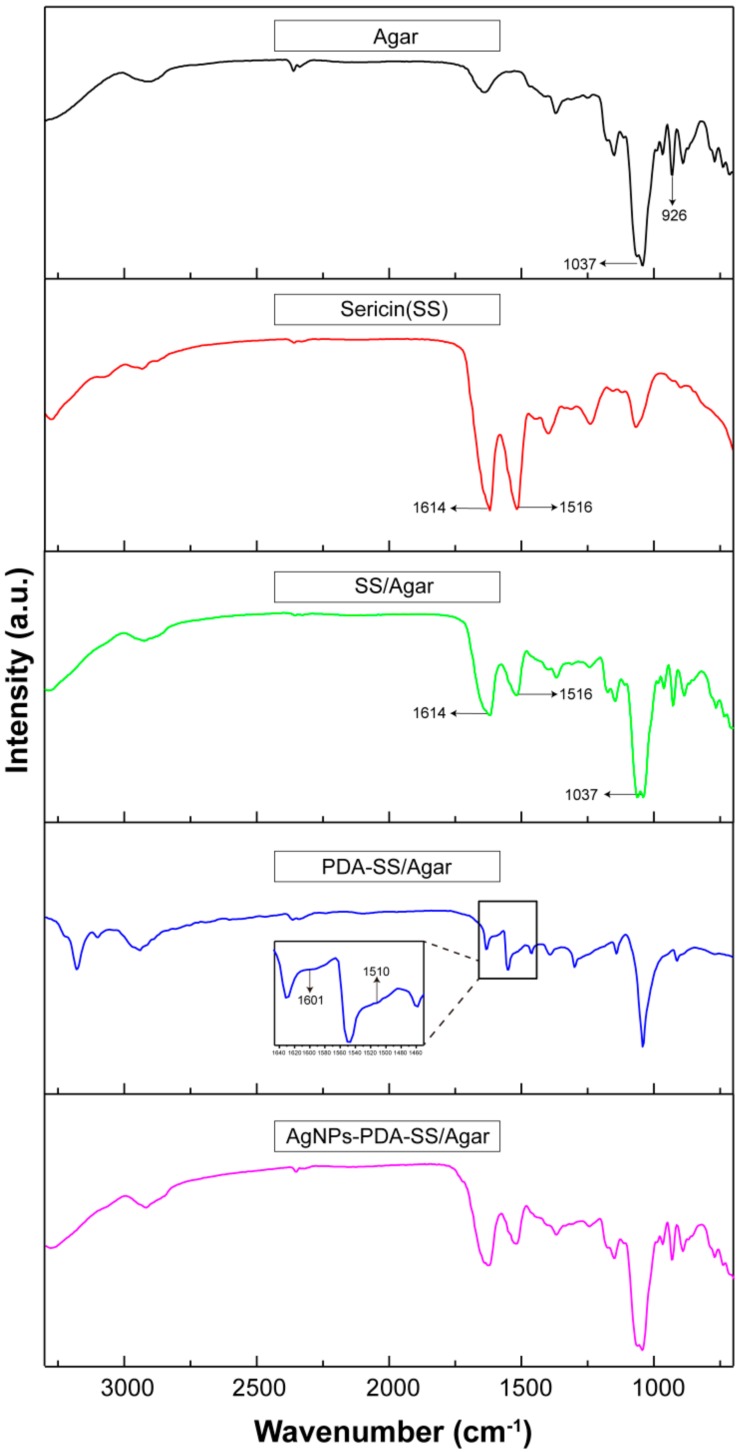
FT-IR spectra of Agar, Sericin (SS), SS/Agar, PDA-SS/Agar and AgNPs-PDA-SS/Agar films.

FT-IR spectra were collected to characterize the structure of different films. The results are shown in Figure 3. The peaks that appeared at 1037 cm^−1^ and 926 cm^−1^ in agar film were characteristic peaks of agar, corresponding to the C=O stretching vibration of 3,6-anhydrogalactose [29]. The two characteristic peaks at 1614 and 1516 cm^−1^ in the sericin film corresponded to amid I and II [30], respectively. Four characteristic peaks at 926, 1037, 1516 and 1614 cm^−1^ occurred in the spectra of SS/Agar, PDA-SS/Agar and AgNPs-PDA-SS/Agar films, indicating the presence of sericin and agar in the blend films, and that the structure of sericin and agar was not affected after blending. After PDA coating, two additional characteristic peaks at 1510 and 1601 cm^−1^ were observed in the spectra of PDA-SS/Agar and AgNPs-PDA-SS/Agar films, which corresponded to the C=C stretching and N-H bending vibrations of the indoline or indole structures in PDA, respectively [31,32]. The appearance of these two peaks demonstrated the successful PDA coating on the SS/Agar film, which was consistent with the observation of SEM (Figure 2b). The spectra of AgNPs-PDA-SS/Agar film showed similar characteristic peaks of sericin and agar with that of SS/Agar film, but differed from that of PDA-SS/Agar film. The possible reason was that the characteristic peak of PDA was close to that of sericin; thus, PDA coating affected the characteristic peaks of sericin and agar, and resulted in the change of SS/Agar spectrum. However, when PDA was used to reduce Ag^+^ to AgNPs, the structure of PDA was changed; thus, the characteristic peak of PDA disappeared and could no longer affect the characteristic peaks of sericin and agar. AgNPs alone did not affect the structure of sericin and agar, and could not change the spectrum of SS/Agar film. 

### 2.3. X-ray Diffraction (XRD)

**Figure 4:**
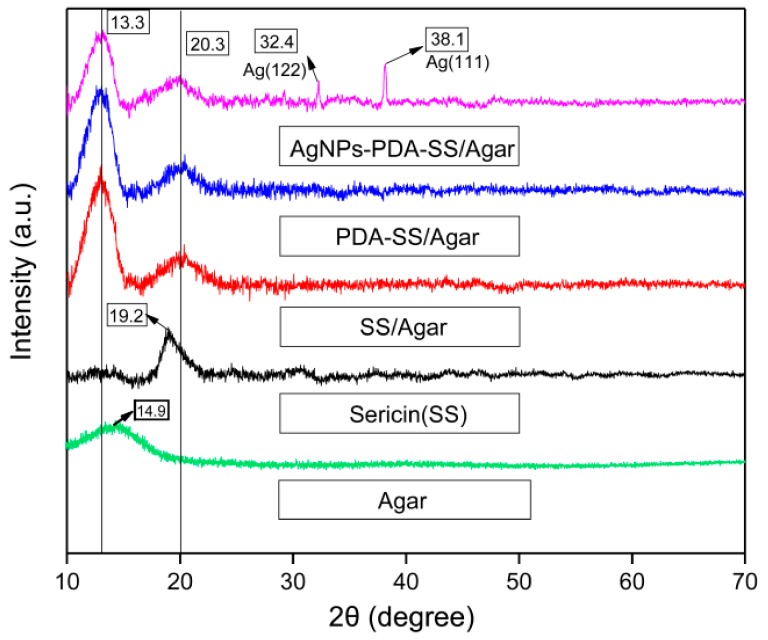
XRD patterns of Agar, Sericin (SS), SS/Agar, PDA-SS/Agar and AgNPs-PDA-SS/Agar films.

The XRD patterns of AgNPs-PDA-SS/Agar, PDA-SS/Agar, SS/Agar, sericin and agar films are shown in Figure 4. The peak located at 2θ = 19.2° corresponded to the silk II structure of sericin protein. The peak at 2θ = 14.9° was the characteristic peak of agar [33]. The peak at 2θ = 13.3° appeared in all XRD patterns except sericin, and was ascribed to the characteristic peak of agar with a slight deviation. The peak at 2θ = 20.3° appeared in all composite films, which may be due to the deviation of the characteristic peak of silk II at 2θ = 19.3° after blending with agar [34]. Our results showed that the blending of sericin and agar slightly changed the characteristic peak of agar and sericin. After PDA coating, the XRD patterns of SS/Agar film did not change, indicating that PDA did not affect the crystal structure of sericin and agar. The diffraction peak at 38.1° and 32.4° could be assigned to the (111) and (122) planes of the face-centered cubic structure of Ag [35,36], demonstrating the presence of AgNPs in the AgNPs-PDA-SS/Agar film.

### 2.4. Wettability and Water Uptake Ability

**Figure 5:**
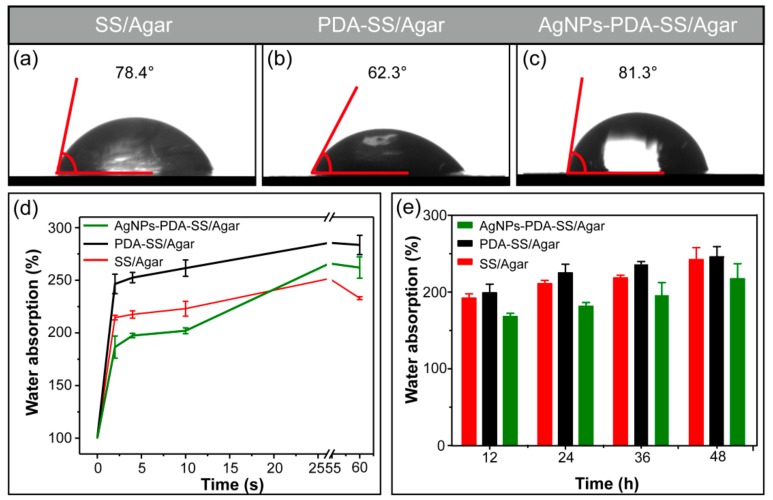
Water contact angle of SS/Agar (**a**), PDA-SS/Agar (**b**), AgNPs-PDA-SS/Agar films (**c**) and water absorption of different films (**d**,**e**).

The water contact angle of SS/Agar, PDA-SS/Agar and AgNPs-PDA-SS/Agar films are shown in Figure 5. The water contact angle of SS/Agar was 78.4°, indicating the surface of SS/Agar was hydrophilic. After PDA coating, the water contact angle decreased to 62.3°, indicating that the wettability of the composite film increased. Figure 5c shows that the water contact angle of AgNPs-PDA-SS/Agar film was 81.3°, suggesting AgNPs modification slightly reduced the wettability of the material surface. The reason may be AgNPs are hydrophobic substances, and the uniform distribution of AgNPs on the PDA-SS/Agar composite film would reduce the hydrophilicity of the film. The water contact angle of AgNPs-PDA-SS/Agar film was still less than 90°, indicating that the prepared material was hydrophilic and potentially useful for biomaterial application.

To further explain the hydrophilicity, the swelling property of the material was tested, as illustrated in Figure 5d,e. The result showed the swelling of AgNPs-PDA-SS/Agar, PDA-SS/Agar and SS/Agar films in 60 seconds. It is obvious that in two seconds, the composite film absorbed a lot of water, indicating that the surface of the composite film had excellent hydrophilicity. The water absorption capacity of PDA-SS/Agar film in a short period of time was better than that of AgNPs-PDA-SS/Agar and SS/Agar films. After 12–48 h, the swelling ratios of different films were in the range of 150% to 250% (Figure 5e), indicating that the prepared film had excellent water uptake ability. According to the moist wound healing theory, moisture promotes wound healing, reduces the pain of dressing removal and scarring and does not destroy freshly formed tissue. Therefore, the excellent hydrophilicity and wetting properties of the prepared composite film are advantageous for wound dressing or other potential biomedical applications.

### 2.5. Mechanical Properties

**Figure 6:**
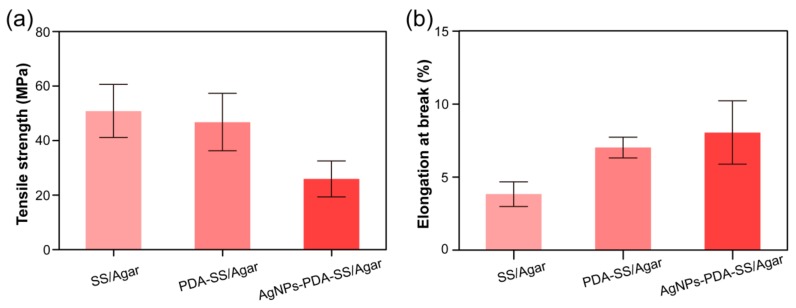
Mechanical properties of different films: (**a**) tensile strength, and (**b**) elongation at break.

Figure 6a,b shows that SS/Agar and PDA-SS/Agar films had tensile strength exceeding 40 MPa. It is known that sericin is fragile and lacks mechanical properties, while agar has high tensile strength [37]. Therefore, the blending of sericin and agar improved the tensile strength of sericin film. The incorporation of AgNPs into PDA-SS/Agar film resulted in the reduction of tensile strength to about 25 MPa, when compared with SS/Agar and PDA-SS/Agar films. This may be because the synthesis of AgNPs on the PDA-SS/Agar film increased the film’s thickness and roughness, which resulted in a reduction in tensile strength. However, this strength was still competent for some applications such as wound caring and food packaging. Elongation at break reflects the flexibility of a material [38]. The elongation at break of SS/Agar was less than 5%. PDA coating increased the elongation at break of SS/Agar film to about 7%, probably due to an increase in the thickness of the composite film. Compared with PDA-SS/Agar film, the elongation at break of AgNPs-PDA-SS/Agar film slightly increased to 8%, indicating the enhanced flexibility of AgNPs-PDA-SS/Agar film. Similarly, thickness and roughness were likely responsible for the increase of the elongation at break of AgNPs- PDA-SS/Agar film. The flexible nature of AgNPs-PDA-SS/Agar film would be beneficial for wound dressing and other potential applications.

### 2.6. Inhibition Zone Assay

**Figure 7:**
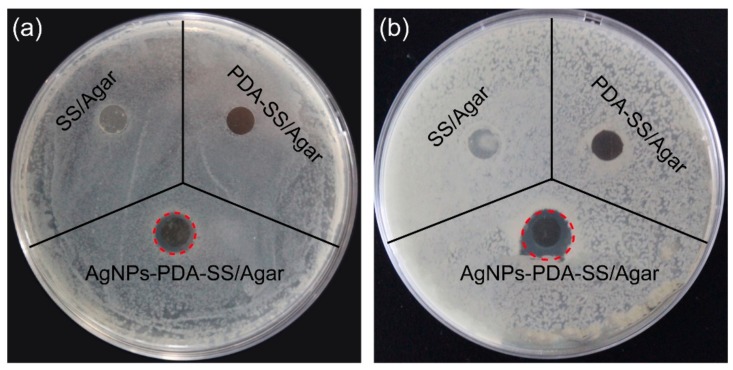
The inhibition zones of SS/Agar, PDA-SS/Agar, AgNPs-PDA-SS/Agar films against *E. coli* (**a**) and *S. aureus* (**b**). Red dotted circle represents the edge of the inhibition zone.

Bacterial infection impedes wound healing. So antibacterial ability is necessary for wound dressing. Figure 7 shows the inhibition zones of AgNPs and PDA-modified or unmodified SS/Agar film against two common bacteria found in wound infections (*S. aureus*, *E. coli*). No inhibition zone appeared for SS/Agar and PDA-SS/Agar films toward the two types of bacteria. An obvious inhibition zone occurred in the presence of AgNPs-PDA-SS/Agar film toward *E. coli* and *S. aureus*, demonstrating that the fabricated AgNPs-PDA-SS/Agar film had excellent antibacterial ability. The diameters of the inhibition zones are shown in Table 1.

### 2.7. Bacterial Growth Curve

**Figure 8:**
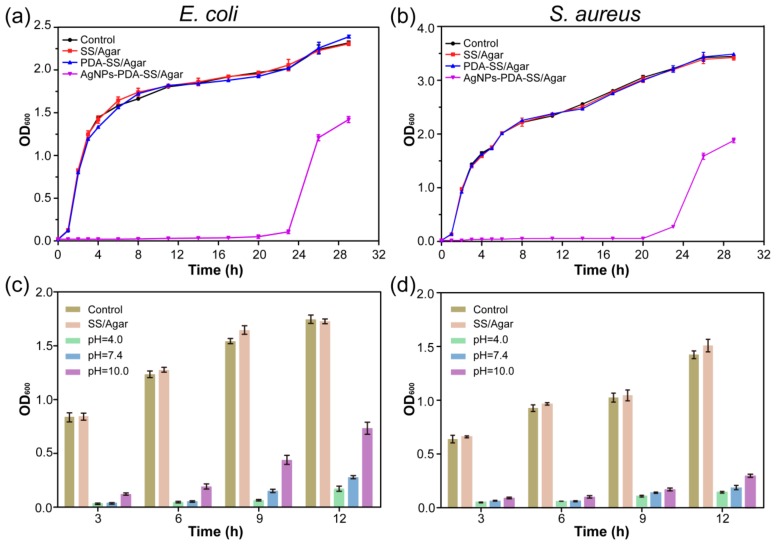
Bacterial growth curve of *E. coli* (**a**) and *S. aureus* (**b**) in the presence of different films, and antimicrobial stability analysis of AgNPs-PDA-SS/Agar film under different pH conditions (**c**,**d**).

A bacterial growth curve experiment was carried out to assess the inhibition effect of AgNPs and PDA treated and untreated SS/Agar film on bacterial growth. Figure 8 shows the growth curves of *E. coli* (Figure 8a) and *S. aureus* (Figure 8b) in the presence of different films, respectively. The growth of *E. coli* and *S. aureus* in the presence of SS/Agar and PDA-SS/Agar films was similar to the control, indicating that SS/Agar and PDA-SS/Agar films did not have bacteriostatic activity. Compared with the control, AgNPs-PDA-SS/Agar significantly inhibited bacterial growth up to 20 h, suggesting that AgNPs-PDA-SS/Agar film had a long-term and efficient inhibition effect on bacterial growth.

### 2.8. Antimicrobial Stability

AgNPs-PDA-SS/Agar film was treated at different pH (4.0, 7.4, 10.0) for 24 h, and then the inhibitory effect of the treated film against *E. coli* and *S. aureus* was determined. As shown in Figure 8c,d, in the absence of AgNPs, there was no significant difference in bacterial growth between SS/Agar and the control at different time points, indicating SS/Agar film had no bacteriostasis ability. Compared with the control, the bacterial growth was obviously inhibited in the presence of AgNPs-PDA-SS/Agar film after treatment with different pH, suggesting AgNPs-PDA-SS/Agar film had stable and long-term antibacterial ability, which was advantageous for wound dressing and other potential applications.

### 2.9. Cytocompatibility

**Figure 9:**
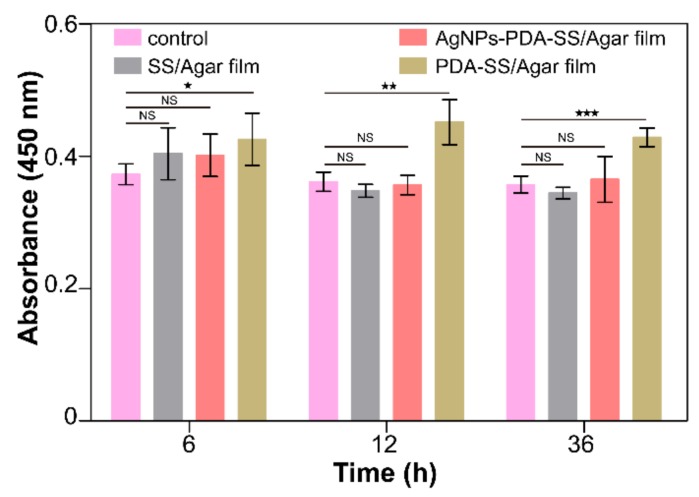
CCK-8 assay of the cytocompatibility of different films on NIH/3T3 cells. The statically significant values are expressed by “NS” (not significant), “★” (*p* < 0.05), “★★” (*p* < 0.01) and “★★★” (*p* < 0.001).

**Figure 10:**
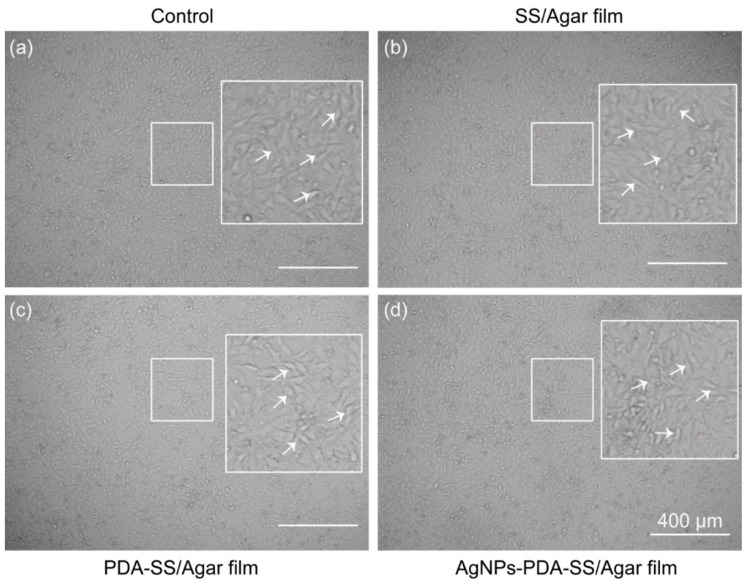
Microscopic observation of NIH/3T3 cells morphology with control (**a**), in the presence of SS/Agar film (**b**), PDA-SS/Agar film (**c**) and AgNPs-PDA-SS/Agar film (**d**). Small box represents a selected area, big box represents the enlarged image in the small box. White arrows indicate the observed fibroblast NIH/3T3 cells. The scale bar is 400 μm.

To evaluate the cytotoxicity of SS/Agar, PDA-SS/Agar and AgNPs-PDA-SS/Agar films, cell counting kit-8 (CCK-8) assay was performed to examine the cells treated with different films. In the test, the metabolically active cells react with the tetrazolium salt in the CCK-8 solution to produce a soluble formaldehyde nitrogen dye with maximum absorbance at 450 nm [39]. Optical density (OD) reflects cell survival and living cells [40]. The results showed there was no significant difference in cell viability between the control and the experimental group treated with AgNPs-PDA-SS/Agar film (Figure 9). Notably, the cell viability when treated with PDA-SS/Agar film was higher than that of the control, indicating PDA was not only non-toxic on cells, but also could promote cell proliferation to improve cell viability. In addition, the cell morphology under different treatments almost did not change after 24 h (Figure 10), suggesting that the prepared films had excellent cytocompatibility on the fibroblast NIH/3T3 cells, which is beneficial for its application in biomaterials.

**Figure 11:**
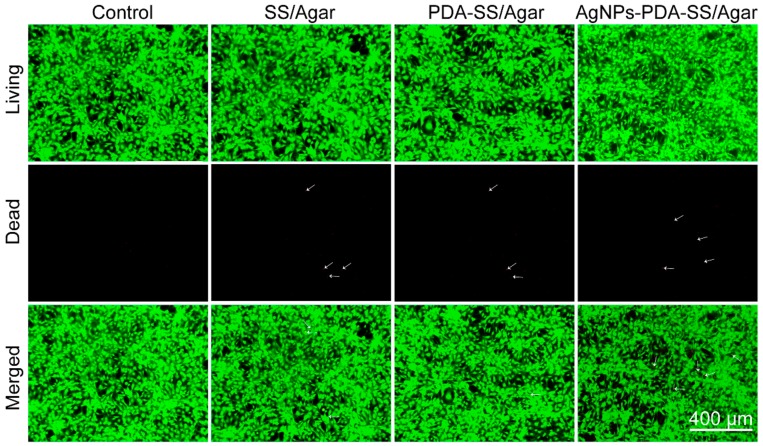
Living/dead cell staining assay of NIH/3T3 cells after being treated with different films. White arrows indicate a very few cells (<1‰) were stained red.

To better visualize the effects of the prepared films on NIH/3T3 cells viability, a living/dead cell staining assay was performed. In this assay, living cells are stained green, while dead cells are red. After being treated with different films for 24 h, the fluorescence images clearly showed almost all cells were stained green, a very few cells (<1‰) were stained red (marked with white arrows, Figure 11), indicating the excellent cytocompatibility of the films on NIH/3T3 cells. This result was in good agreement with that of CCK-8 assay and the microscopic observation on cell morphology.

## 3. Materials and Methods

### 3.1. Materials and Chemicals

The strain of silkworm we used in this work was a commercial silkworm strain 872. Silkworms were reared in our laboratory with fresh mulberry leaves at 25 °C and 75% relative humidity under 12 h photoperiod. Fresh silkworm cocoons were collected for sericin preparation. Dopamine hydrochloride and silver nitrate (AgNO_3_) were purchased from Aladdin (Shanghai, China). Hydrochloric acid and Tris (hydroxymethyl) aminomethane (Tris) were from Sangon Biotech (Shanghai, China). Ultrapure water made by a MilliQ water purification system (Millipore, Billerica, MA, USA) was used in the experiment. The Cell counting kit-8 (CCK-8) used in the experiment was bought from Beyotime (Beijing, China). LIVE/DEAD cell viability kit was bought from Thermo Fisher Scientific (Waltham, MA, USA). NIH3T3 (mouse embryonic fibroblast) cell lines were received from the China Infrastructure of Cell Line Resources. The Dulbecco’s modified Eagle’s medium (DMEM), Penicillin/Streptomycin, Fetal Bovine Serum (FBS), and Trypsin-EDTA were bought from Gibco BRL (Gaithersburg, MD, USA).

### 3.2. Preparation of AgNPs-PDA-SS/Agar Film

Sericin was obtained from silkworm cocoons through high temperature. Silkworm cocoons were cut into pieces and treated at 121 °C and 0.1 MPa for 15 min. Then, sericin was extracted into solution and separated from silk fibroin by filtration, which is descripted in our previous report [41,42,43]. Agar was dissolved in water with agitation at 90 °C to a final concentration of 2% (*w/v*). Sericin solution (2%, *w/v*) and agar solution (2%, *w/v*) were well mixed as a ratio of 1:1 at 60 °C. Subsequently, the mixture was dried at 65 °C overnight to form SS/Agar film. Next, dopamine hydrochloride was dissolved in Tris-HCl buffer (pH 8.5) to form polydopamine solution (2%, *w/v*), the chemical process was shown in Figure 1b. SS/Agar film was directly placed in freshly prepared polydopamine solution with stirring at 37 °C for 12 h. Then, the PDA-treated blend film was removed, washed by water for three times, and dried at 25 °C for 12 h to obtain PDA-coated SS/Agar composite film. Next, PDA-SS/Agar film was immersed into AgNO_3_ solution (10 mM) at 25 °C for 4 h. After multiple washes, the film was dried at 25 °C to yield AgNPs modified PDA-SS/Agar film. The average thickness of the SS/Agar, PDA-SS/Agar and AgNPs-PDA-SS/Agar films determined by microscope were 145.47 μm, 153.29 μm and 158.90 μm, respectively.

### 3.3. Material Characteristics

The surface morphologies of SS/Agar, PDA-SS/Agar, AgNPs-SS/Agar and AgNPs-PDA-SS/Agar films were characterized by JEOL scanning electron microscopy JSM-6510LV (Tokyo, Japan). The films were cut into strips with a dimension of 1 cm × 1 cm (length × width), dried, and sputtered with gold prior to SEM test. The accelerating voltage for the test was 20 kV. The working distance was 10 mm. Energy dispersive spectra (EDS) were collected on Oxford INCA X-Max 250 (Abingdon, UK) during SEM test to analyze the chemical elements. XRD spectra were recorded on PANalytical x’pert (Almelo, Netherland) within 10–80°. Nicolet iz10 FT-IR spectrometer (Thermofisher Scientific, Waltham, MA, USA) was used to obtain FT-IR spectra in the wavenumber of 4000–800 cm^−1^.

### 3.4. Hydrophilicity

The hydrophilicity of the prepared films was measured by surveying the sessile drip contact angle using a KRÜSS DSA100 contact angle analyzer (Hamburg, Germany) at 25 °C. Water droplets (4 μL) were dispensed on the surface of the film and the water contact angle was measured. Five points are measured for each sample and averaged.

### 3.5. Water Absorption Ratio and Moisture Retention Capacity

Water absorption ratio was used to characterize the swelling property of the film. The dry films (3 cm × 3 cm, length × width) were weighed, and then immersed into water. Afterwards, the swollen samples were removed from water at different intervals and weighed after the removal of water on the surface by filter paper. Swelling property was defined as follows:

Water absorption ratio (%) = (m_2_ − m_1_)/m_1_ × 100% (1)

where m_1_ and m_2_ were the weights of dry and swollen films, respectively. Three replications per sample were made to ensure the accuracy of the experiment.

### 3.6. Mechanical Properties

For film materials, the tensile strength and elongation at break are two common indicators for mechanical properties. The tensile strength and elongation at break of the films were studied by SHIMADZU AG-X plus (Tokyo, Japan), which is a common instrument for mechanical properties analysis. For the test, the films with a dimension of 4 cm × 1 cm (length × width) were measured with a crosshead speed of 3 mm/min. The length and width of the samples were determined according to the mold we used, and the stretching rate was suggested by the manufacturer’s protocol and validated by previous reports [44,45,46]. At least 8 replicates of an individual film were examined.

### 3.7. Inhibition Zone Assay

The method presented by Schillinger and Lücke was applied to test anti-bacterial inhibition zone of the prepared films [47]. Briefly, *E. coli* and *S. aureus* were inoculated into Luria-bertani (LB) medium (pH 7.4) with shaking at 37 °C until optical density (OD) value at 600 nm (OD_600_) reached 1.0. Then, bacterial suspension (200 μL) was uniformly spread on agar plate in the presence of SS/Agar, PDA-SS-SS/Agar and AgNPs-PDA-SS-SS/Agar films and incubated at 37 °C for 24 h. The diameters of inhibition zone around the samples were measured.

### 3.8. Growth Curve Assay

The growth curve analysis was carried out based on Pal’s protocol [48]. Bacteria were inoculated into LB medium and cultured at 37 °C with constant shaking (220 rpm) in the presence of different films. Then, bacterial suspension (0.5 mL) was collected at different time intervals to measure OD_600_. All samples were tested in triplicate.

### 3.9. Antimicrobial Stability

To test the antibacterial stability of AgNPs-PDA-SS/Agar film, the films (1 cm × 1 cm, length × width) were soaked in PBS buffers (pH 4.0, 7.4, 10.0). After 24 h, the films were dried at 25 °C and then added into 10 mL bacterial suspensions with the same initial OD_600_. Bacterial suspensions (0.5 mL) were collected at different intervals and OD_600_ was measured to determine the antibacterial stability of the prepared films.

### 3.10. Cytotoxicity

The fibroblast NIH/3T3 cells were cultured in Dulbecco’s modified eagle medium at 37 °C with 5% CO_2_ and 95% relative humidity. Prior to the cytotoxicity test, the circular SS/Agar, PDA-SS/Agar and AgNPs-PDA-SS/Agar films (diameter = 7 mm) were irradiated by UV light overnight to ensure the sterility of the materials. NIH/3T3 cells (100 μL) were loaded in a 96-well plate at a density of 1 × 10^4^ cells/well and incubated at 37 °C for 12 h in the presence of the sterile films. Untreated cells were set as controls. CCK-8 kit was used to detect cells viability after treated with different films. After various time, the films were removed from the medium, and CCK-8 solution (10 μL) was added into each well and incubated at 37 °C. After 1 h, the optical density (OD) were measured at 450 nm on a Tecan Infinite M200 Pro microplate reader (Männedorf, Switzerland). Cell viability was the percentage of OD values in the treated and control groups. Each experimental group was tested for at least three replications. After culture for 24 h, NIH/3T3 cells morphology were observed on a fluorescence microscope.

In addition, a living/dead cells staining assay was performed to further assess the effect of the films on NIH/3T3 cells. NIH/3T3 cells were cultured at 37 °C as mentioned above. After being treated with SS/Agar, PDA-SS/Agar and AgNPs-PDA-SS/Agar films for 24 h, the staining solution (30 μL) was added into each well and incubated with the cells at 37 °C for 15 min. The fluorescence images were recorded on an Invitrogen EVOS FL Auto Cell Imaging System (Waltham, MA, USA). Each sample was tested in triplicate.

### 3.11. Statistics

All experiments were performed at least in triplicate, and the results were presented as average ± standard deviation (SD). Student’s *t*-test, together with variance analysis, was carried out to determine the statistical significance between two groups. The statically significant values were expressed by “★” (*p* < 0.05), “★★” (*p* < 0.01) and “★★★” (*p* < 0.001). 

## 4. Conclusions

In this study, we successfully synthesize high-density AgNPs on PDA-SS/Agar composite film with the assistance of PDA. AgNPs-PDA-SS/Agar composite film exhibits good hydrophilicity and proper mechanical properties. In addition, AgNPs-PDA-SS/Agar film has high efficiency and durable antibacterial ability, and excellent compatibility on the fibroblast NIH/3T3 cells. These excellent properties facilitate the potential applications of AgNPs-PDA-SS/Agar film in wound dressing, tissue engineering and antibacterial packaging.

## Figures and Tables

**Table 1:** Diameters of the inhibition zones of SS/Agar, PDA-SS/Agar and AgNPs-PDA-SS/Agar films against *E. coli* (**a**) and *S. aureus* (**b**).

Bacteria	SS/Agar (cm)	PDA-SS/Agar (cm)	AgNPs-PDA-SS/Agar (cm)
*E. coli*	1.10 ± 0.00	1.10 ± 0.00	1.63 ± 0.03
*S. aureus*	1.10 ± 0.00	1.10 ± 0.00	1.91 ± 0.11
